# Bioengineered Scaffolds as Substitutes for Grafts for Urethra Reconstruction

**DOI:** 10.3390/ma12203449

**Published:** 2019-10-22

**Authors:** Martina Culenova, Dusan Bakos, Stanislav Ziaran, Simona Bodnarova, Ivan Varga, Lubos Danisovic

**Affiliations:** 1 Institute of Medical Biology, Genetics and Clinical Genetics, Faculty of Medicine, Comenius University, Sasinkova 4, 811 08 Bratislava, Slovakia; 2 International Centre for Applied Research and Sustainable Technology, Jamnickeho 19, 841 04 Bratislava, Slovakia; dusan.bakos@stuba.sk; 3 Department of Urology, Faculty of Medicine, Comenius University, Limbova 5, 833 05 Bratislava, Slovakia; stanoziaran@gmail.com; 4 Department of Biomedical Engineering and Measurement, Faculty of Mechanical Engineering, Technical University of Kosice, Letna 9, 042 00 Kosice, Slovakia; simona.bodnarova@gmail.com; 5 Institute of Histology and Embryology, Faculty of Medicine, Comenius University, Sasinkova 4, 811 08 Bratislava, Slovakia; 6 Regenmed Ltd., Medena 29, 811 01 Bratislava, Slovakia

**Keywords:** artificial urethra, scaffolds, electrospinning, bioprinting

## Abstract

Urethral defects originating from congenital malformations, trauma, inflammation or carcinoma still pose a great challenge to modern urology. Recent therapies have failed many times and have not provided the expected results. This negatively affects patients’ quality of life. By combining cells, bioactive molecules, and biomaterials, tissue engineering can provide promising treatment options. This review focused on scaffold systems for urethra reconstruction. We also discussed different technologies, such as electrospinning and 3D bioprinting which provide great possibility for the preparation of a hollow structure with well-defined architecture.

## 1. Introduction

Urethral defects, as a consequence of congenital malformations, trauma, inflammation or carcinoma, still pose great challenges to urology. Hypospadias represents the most common birth defect of the urethra characterized by displacement of the urethral meatus, penile curvature, and less developed foreskin. Various surgical techniques are used for the management, but, generally, surgical intervention consists of several steps: penile degloving, correction of the ventral curvature, urethroplasty, glansplasty, and cosmetic skin coverage. Options for the urethroplasty include urethral plate tubularization, augmentation or replacement. Esthetics and functionality are the main goals to be achieved by proper treatment. Patients undergo surgery between 6 and 18 months of age. In western countries, the incidence of this disease is 0.4%–0.6% [1]. In adults, urethral strictures occur with high frequency [2]. The main cause is related to iatrogenic injuries as a result of diagnostic or therapeutic procedures which the patient undergoes and are caused by a physician [3]. In the context of urethral damage, it is mainly traumatic catheterization or long-term insertion of an indwelling catheter which causes short- and long-term complications due to the ischemia of the urethral tissue. Short-term complications can be present as acute urine retention, urosepsis, bleeding, and acute kidney injury. Urethral stricture occurs among the long-term complications, and iatrogenic injury is believed to be counted for 33% of the causes in the developed world [4]. Tortuous anatomy and obstruction of the lower urinary track can be predisposing factors for tissue damage [5]. Urethral stricture is described as the narrowing of the urethral lumen due to the presence of tissue scarring. Symptoms are derived from the obstruction of the lower urinary tract, e.g., poor stream, incomplete voiding, urinary retention, etc. In many cases, a surgical procedure, which consists of damaged tissue removal, presents the only option to resolve this problem. More than 300 surgical approaches are available. End-to-end anastomosis is often used to repair short (<1 cm) and simplex strictures. This group of the strictures can be well managed by current surgical techniques. In spite of this fact, patients often have to undergo repeated intervention due to the fact of stricture reoccurrence [6]. The replacement of complex and long strictures is more challenging. When managed by common surgical procedures, long-time outcomes are not satisfactory as the rate of the stricture reoccurrence is very high. The use of the tissue graft or flap (from buccal mucosa or penile skin) is a widely adopted technique used for long-segment and complex urethral strictures. However, donor site morbidity complications remain a big issue (e.g., bleeding, hematoma, and nerve damage) [7]. In respect to the abovementioned issues, tissue engineering may provide promising treatment options. The main goal is to create a functional tissue substitute using a limited amount of material (e.g., cells, scaffolds) under in vitro conditions.

Tissue engineering is a rapidly emerging branch of medical science that provides solutions by creating conditions for the replacement and/or repair of native tissues that are diseased, damaged or lost. Generally, three different methodological approaches can be distinguished: cell-based therapy, the use of biomaterials or acellular scaffolds, and bioactive molecules [8]. Numerous types of scaffolds have been described and used in the regeneration of different tissues and organs in the body. Tissue engineering has found important applications in the field of clinical urology, and the use of scaffolds, in particular, is a promising tool for urethral reconstruction. From initial tissue substitution using simple biological scaffolds to reconstruction with a combination of seeded cells and scaffolds, the application of tissue engineering technology to urethral reconstruction is a significantly developing field [9,10].

This review focused on the use of different scaffold systems for urethral reconstruction in cases where grafts are used currently. Specifically, the materials used and the different manufacturing techniques as well as the potential for successful use clinically is covered. It has already been proved that cell-seeded constructs are superior when compared with acellular matrices [11]. The scaffolds act as a supporting spine for the reconstruction of tissues of the outer and inner wall of the urethra while retaining structural stability and allowing tissue growth in 3D space. The most important aspects in scaffold design are biocompatibility, biodegradability, applicable mechanical properties, scaffold structure, architecture, and manufacturing technology. Various types of biodegradable and non-biodegradable scaffolds have been developed for urinary tract tissue engineering applications [12]. These biomaterials can be manufactured from polymers of natural and synthetic origin, which may be modified to prepare appropriate scaffolds with defined biological and biomechanical characteristics. Recent studies utilizing cell-seeded natural biomaterials in urethral repair have documented promising results. However, new approaches in tissue engineering that make use of cell-seeded synthetic materials hold the possibility of even better results [13].

A scaffold from biological or synthetic materials can be used without cell-seeding; it is expected that adjoining cells will gradually incorporate into the scaffold. This approach has limitations due to the possibility of cells overgrowing. Another limitation is the fact that cells from surrounding tissues can migrate onto the scaffold within a distance of 0.5 cm [11]. In the case of long urethral segment repair, it is necessary to seed the scaffold with autologous or stem cells, because the ingrowth of cells from adjacent tissue is not fast enough. This approach has the potential for improving the success rate and reducing complications in surgical reconstruction of the urethra compared to using grafts [14].

Recently, there are many technological approaches (e.g., electrospinning and bioprinting) that can be used to prepare scaffolds as substitutes for grafts with well-defined structural features as well as biomechanical characteristics (e.g., structural strength, porosity, biodegradability, etc.) [15].

In general, bioartificial tissues must be biocompatible and well-vascularized [16]. Structural integrity and mechanical stability are necessary for surgical manipulation with these substitutes. Although strength is necessary, the material must also comply with the requirements of elasticity and flexibility. The process of biodegradation during tissue remodeling should be synchronized with the speed of the tissue ingrowth. Vascularization of the tissue-engineered scaffolds, via angiogenesis, will control the rate of tissue ingrowth but also must be fast enough to prevent seeded cells from dying. If all of these requirements are met, the engineered tissue may successfully support organ/tissue reconstruction, as shown in a study by Raya-Rivera et al. (2011) [17]. This study described the transplantation of the cell-seeded polylactide-*co*-glycolide (PLGA) graft to five patients with urethral defects. Their results provided evidence that tissue-engineered scaffolds have promise for urethral stricture repair.

## 2. Biological Scaffolds

### 2.1. Acellular Matrices

A great number of studies on scaffolds for urethral reconstruction have addressed the use of autologous sources. Within this context, biological materials are usually understood to be matrices derived from urinary tract tissue, the submucosa of the small intestine [18,19], the submucosa of the bladder [20] or buccal mucosa [21], etc. Acellular tissue matrices have an important position in tissue engineering, as they can mimic the extracellular matrix effectively [22].

Human amniotic membrane (hAM) and hAM-derived cells possess properties (low immunogenicity, promotion of epithelization, anti-inflammatory properties, angiogenic and antiangiogenic properties, antifibrotic properties, antimicrobial properties, and anticancer properties) that make them suitable for regenerative medicine. Ramuta et al. [23] confirmed the potential of hAM for use in reconstructive urology. However, they also advised of concerns with such use, including the lack of a standardized protocol in hAM preparation and storage, heterogeneity of hAM, and especially hAM’s low mechanical strength.

Collagen constructs are very popular in tissue engineering. Such constructs derived from the bladder submucosa (lamina propria) have also been used as substrates for urethral repair, either with or without cells [24,25]. Collagen-based matrices are biocompatible, flexible, and easily prepared in large quantities. Dorin et al. [11] used unseeded matrices derived from the porcine bladder in a study where they estimated the maximum distance over which natural cells can regenerate damaged tissue over such a scaffold. The scaffolds were tubularized around a 16Fr catheter (outer diameter 5.333 mm) with lengths from 0.5 to 3.0 cm. Defects of 0.5 cm length were repaired without strictures, but at longer lengths, cellular ingrowth was present only at the sites of the anastomosis with increased deposition of collagen and fibrosis in the center of the graft.

Jia et al. [26] developed a collagen scaffold modified with a collagen-binding vascular endothelial factor (VEGF) (which is a fusion protein of VEGF joined with a collagen-binding domain, also referred to as CBD-VEGF) to replace long urethral segments (5 cm) in a canine model. Collagen membranes of the size 5.0 cm × 3.0 cm × 0.2 cm were tabularized during the surgery by continuous suture with synthetic absorbable suture according to the diameter of the host urethra. The CBD-VEGF was dissolved in PBS (phosphate buffered saline) and the collagen scaffold was soaked with this solution until implantation. Upon comparison of results with modified and unmodified scaffolds, the group with collagen-modified scaffolds showed better epithelial formation, significant re-vascularization, and better smooth muscle regeneration. The main aim of this study was to establish whether unseeded but modified scaffolds are applicable for long urethral defect repair.

Double-layered high-density collagen gel tubes were prepared in a study by Pinnagoda et al. [27]. Collagen tubes were prepared from sterile rat-tail type I collagen in minimum essential medium and neutralized with NaOH. The gel substance was molded into tubular shapes with the desired parameters. After a morphology study of these scaffolds, the tubes were implanted into a rabbit model to repair 2 cm long urethral defects. The regenerative abilities of these gel tubes were evaluated postoperatively. The results showed time-dependent, spontaneous urethral and smooth muscle tissue regeneration; however, 20% of the animals developed complications (stenosis, fistulae). The significance of this study is that it challenged previous statements about the ability of unseeded scaffolds to repair defects only up to 0.5 cm. Moreover, the double-layered scaffolds enable better surgical handling; tubes can be sutured with no need for synthetic polymers to be present in their structure. 

Larsson et al. [28] prepared tubular cell-free collagen matrices with different collagen density and fiber distribution thus resulting in a polarized low fiber density collagen graft (LD-graft). As a control, the authors also generated a uniform high fiber density collagen graft (HD-graft). Both grafts were implanted to bridge a 2 cm long urethral defect in a rabbit model. According to the histological evaluation, the LD-graft had better smooth muscle regeneration compared to the HD-graft. The goal was to study how the density of collagen fibers influences the success of the urethral repair in vivo. The authors wanted to specify that acellular grafts with high stiffness and density are not effective for tissue repair. 

### 2.2. Cell Loaded Matrices

Orabi et al. [24] investigated the possibility of using collagen-based seeded scaffolds to design long urethral segments in canines, a more clinically relevant animal model. Autologous bladder epithelial and smooth muscle cells from male dogs were seeded onto collagen-based tubular matrices (6 cm in length). While urethral segments replaced with acellular scaffolds collapsed, urethral substitutes with cells showed normal tissue formation without evidence of fibrosis. The reason for the collapse is probably due to the inability of the cells from native adjacent tissue to migrate further than the edges when it comes to long urethral defects. Therefore, the use of cell-seeded scaffolds seems to be more promising. Reconstruction of long urethral defects using collagen matrices was the subject of a study by El-Tabey et al. [29]. Matrices were harvested by bladder excision of female dogs. Autologous urothelial and smooth muscle cells were used to create a cell-seeded construct to repair a 3 cm long defect. The results were not satisfactory, as histopathological examinations revealed insufficient vascularization and developed fibrosis in the substitutes. The urethral stricture was observed in all dogs included in this study. The authors attribute this phenomenon to an exaggerated immune response which is known to exist in street mongrel dogs (these were chosen for the animal model). 

In an interesting biomaterials study by Micol et al. [30], collagen tubes were used which had been made by molding of acid-soluble rat-tail collagen neutralized with NaOH. To obtain a sufficiently strong scaffold, nylon mesh and tissue papers were wrapped around the tubular hydrogel, and water was then extracted. Both acellular and cell-seeded tubular substitutes (2 cm long) were implanted into rabbits. The length of the urethral defect was 1 cm. The histology of acellular and cell-seeded substitutes showed spontaneous regrowth of urothelium which, according to the authors, might be clinically useful as an effective treatment for congenital and other urethral pathologies. Moreover, the authors highlighted the short time interval after which the engineered substitutes were implanted. The period was 24 hours. 

In a study by Zhu et al. [31], epidermal growth factor (EGF) and mitomycin C (MMC) were loaded into a tubular gelatin scaffold that was prepared by freeze drying in a specific mold. The composition and fabrication methods were designed to provide time-dependent controlled release of EGF and MMC. The degree of crosslinking (genipin used as a natural crosslinker) and scaffold properties, biodegradation, and in vitro release tests were evaluated. Mitomycin C was loaded into polylactide microspheres by emulsion solvent evaporation. Silk fibroin was used as a carrier for EGF. Tests for MMC and EGF release were performed with spectral methods. Urethral epithelial cells and urethral scar-derived fibroblasts harvested from male adult rabbits were seeded on modified and unmodified scaffolds. The result showed that mechanical properties and degradation depended on the rate of crosslinking and that release of EGF occurred early while MMC was released later. The growth of the urethral scar-derived fibroblasts was inhibited. 

Cell-seeded matrices have been recommended for urethral reconstruction in a study that focused on the effectiveness of long segmental penile urethral replacement with an autologous cell-seeded tubularized collagen-based matrix. Urethrograms showed that male rabbits implanted with such substitutes maintained a wide urethral inner diameter without strictures [23]. In another study, acellular collagen matrices were generated from allogeneic rabbit bladder submucosa. Isolated autologous foreskin epidermal cells from rabbits were expanded in vitro and labelled with 5-bromo2’-deoxy-uridine (BrdU). The labelled cells were seeded onto tubular acellular collagen matrices (1.5 cm × 1 cm). Over several months, epidermal layers with abundant vessels in the submucosa were formed [32]. 

Scaffolds with the properties of shape recovery and radial elasticity were prepared in a study by Versteegden et al. [33]. Swelling, homogenizing, freezing, and freeze drying of insoluble collagen suspensions were steps taken in preparing these tubular collagen scaffolds. Collagen suspensions were poured into a polypropylene mold with a centered steel mandrel and compression and carbodiimide crosslinking were applied to obtain thin, tubular collagen sheets with star-shaped lumens. After the characterization of morphology and mechanical testing, squamous bladder carcinoma cells were seeded onto the scaffolds under static conditions; later, the seeded scaffolds were transferred to a dynamic flow bioreactor system. The results showed that such engineered substitutes successfully displayed elastic-like characteristics. This type of scaffold was described as the first self-expandable tubular implant consisting solely of type I collagen [34].

Silk fibroin (SF) is a protein and well-known natural biomaterial that has the potential for use in urethra reconstruction. Previous studies have shown that silk fibroin scaffolds from an aqueous system degraded completely in vivo within 2–6 months [35]. This length of degradation is considered suitable for urethra reconstruction. Lv et al. [36] studied modified silk fibroin/keratin films with improved mechanical properties when blended with gelatin. They also investigated the incorporation of an oxygen-generating substance, calcium peroxide, into the blended films. They report that the incorporation of calcium peroxide (CPO) into a scaffold composed of human keratin, silk, and gelatin results in a new type of oxygen-generating substitute. Oxygen generation from these scaffolds was analyzed to determine optimal CPO concentrations. Mechanical properties and morphology of the scaffolds were also evaluated. High levels of released oxygen were maintained over two weeks. To estimate cytotoxicity and in vivo behavior, rabbit smooth muscle cells were seeded onto the scaffolds and these cell-seeded constructs were then applied to a rabbit model of ventral urethral defect. The phenomenon of oxygen release caused a slight decrease in mechanical properties. But on the other hand, enhanced cell proliferation was observed compared to unmodified scaffolds. However, mild fibrosis of urethra was still detected in experimental group. Although Lv’s study used a film form of this scaffold, tubularisation for urethra reconstruction is possible.

Table 1 provides an overview of materials which were used for fabrication of tubular urethral tissue substitutes.

## 3. Biodegradable Synthetic Scaffolds

Synthetic biodegradable scaffolds can be made from various polymers with widely ranging morphologies and structural properties. Potential advantages of using such synthetic polymers include the elimination of any risk of viral disease transmission and the ability to custom-design scaffolds to individual patient considerations. On the other hand, there are certain requirements that synthetic scaffold materials must meet: they must be biodegradable and the rate of scaffold degradation in vivo should be synchronized with tissue regeneration. The scaffolds should also predictably support urethral reconstruction and have appropriate mechanical strength. Additionally, if they are to be used in clinical applications, such synthetic materials must be produced aseptically, or they must be capable of being made sterile. Importantly, synthetic materials offer both reproducibility and the possibility of incorporating signal molecules into the scaffold structure. Compared with biological scaffolds, synthetic scaffolds also have an advantage in their ability to resist immunological rejection when implanted without cells or seeded with autologous cells. Such synthetic biodegradable polymers as poly-lactones, including poly-lactic acid (PLA), poly-glycolic acid (PGA), and poly-caprolactone (PCL), and their copolymers are commonly studied and have been used for biomedical devices due to the fact of their documented biocompatibility. In particular, poly-lactic acid (PLA) is considered one of the most generally promising biodegradable polymers, as it possesses the required mechanical and biological properties and good thermoplastic processing. The rate of PLA-based scaffold degradation depends on structural rigidity related mostly to their crystallinity [9]. 

The aim of the research by Dorati et al. [37] was to investigate the applicability of a graft copolymer of PLA, termed LMP-3055, in the biological environment of tissues and its capability of use in tissue engineering. The copolymer was synthesized to improve the toughness and tensile properties of the related PLA homopolymer, which has limited applications in tissue engineering due to the fact of its brittle nature. PLA, poly glycolic acid, and their co-polymers, and poly lactic-*co*-glycolic acid have been used to treat urethral strictures in preclinical or clinical settings. However, the ideal matrix for their use in urethroplasty remains controversial. For example, P96L/4DLA is available commercially from Scaffdex (Tampere, Finland) as a film or as a braided tubular stent. They have the required biological properties and studies have shown their suitability when implanted in humans, rabbits, and mice [38]. Selim et al. [39] have studied how sterilization and cell seeding techniques affect the physical properties of the scaffolds. The goal was to evaluate how these steps might change the quality of tissue-engineered buccal mucosa when compared to the native tissue. In conclusion, the authors showed that PAA (peracetic acid) and γ-irradiation sterilization techniques are suitable for PLGA (polylactic-co-glycolide) scaffold sterilization, in spite of a reduction in the tensile properties. Nevertheless, cells grew well on the sterilized scaffolds. They concluded that sterilized PLGA 85:15 has the potential for preparation of the tissue-engineered buccal mucosa.

A wide range of urethral properties must be taken in account when designing a tissue engineering scaffold, from the location of repair in the urethra to the sex of the individual. The ability to regulate the mechanical properties of a scaffold according to such biological variables is thus an important consideration. One way to approach these concerns during scaffold design is to switch from a hard-tough polymer (e.g., PLA) to a highly elastomeric one, such as a polyurethane. Polyurethanes (PEUs) have a long history of use in medical applications and their mechanical properties can be tuned to specific applications, as well. Different studies have reported that the degradation products are non-cytotoxic, as reviewed in Reference [40]. Polyurethane scaffolds have excellent mechanical properties that can be controlled by changing the molar ratio of the initial components, and they can be used for specific applications where elasticity and strength are required. Polyurethanes can be molded into tubes, a highly desirable property when urethral reconstruction is required. Hicks et al. [41] investigated hybrid materials consisting of two synthetic biodegradable polymers, porous polyurethane-polycaprolactone (PEU-PCL) and braided tubes of P96L/4DLA. The authors detected three types of urethral adhesive proteins (type I collagen, type IV collagen, and vitronectin) which can ease cell colonization of the scaffolds. Moreover, these proteins may be related to the anchoring of the epithelial layer to the basal membrane. Their use might be helpful for the engineering of a urethral substitute with sufficient barrier function.

In a clinical trial, Raya-Rivera et al. [16] treated five boys with posterior urethral defects. A tissue biopsy was taken from each patient. Autologous muscle and epithelial cells were seeded onto tubularized polyglycolic acid:poly(lactic-co-glycolide acid) scaffolds for urethral reconstruction. Posterior urethroplasty was performed. The result of the surgical procedure was evaluated by signs of the absence or presence of dysuria, voiding frequency, straining, and dribbling. Each patient who underwent cystourethroscopy, uroflow studies, biopsy sampling, and voiding cystourethrograms were evaluated, as well. The authors concluded that tubularized urethra can be engineered and remain functional in a clinical setting for up to 6 years. Repair of long urethral defects often requires long graft tissues and extensive surgery, which increases the requirements that must be met when choosing a scaffold. 

There are other methods of preparing porous, tubularized, biodegradable scaffolds which are worth mentioning, even if not all have yet seen clinical use. In addition to conventional techniques, additive manufacturing or 3D printing can be applied to design tubular scaffolds. During additive manufacturing, the scaffold is created layer by layer until its final form. It is possible to use a variety of materials for scaffold fabrication with this technique allowing for the control of the microarchitecture with much higher precision, as the entire process is supported by computer software (computer-aided design, CAD). The aim is to mimic the original structure of the tissue or organ. Implementation of CAD models into direct production of scaffolds is presented as computer-aided tissue engineering [42]. 

Porous materials can be produced by salt particulate leaching [43,44] or by phase separation [45] or micro-structuring techniques [46]. The pore size and degree of porosity vary, but these techniques result in materials with macro-porous structures that are usually from 10–500 μm. On the other hand, these techniques include several preparation steps which make them work-intensive and time-consuming.

Table 2 provides an overview of biodegradable synthetic scaffolds used as tubular urethral substitutes.

## 4. Electrospinning

Interest in electrospinning has recently escalated, especially due to the technique’s ability to produce biomaterials with nanoscale properties in the field of medicine. This scaffold construct technology is focused on the design, manufacture, and characterization of three-dimensional (3D) scaffolds for cell seeding using in vitro or in vivo culturing. A non-woven matrix of electrospun nanofibers possesses the high porosity and spatial interconnectivity necessary for tissue engineering applications [47]. Novel strategies in electrospinning technology include using a variety of scaffold compositions and architecture, along with the addition (encapsulation) of cells and biologically active substances within the scaffold [48]. Improved deposition efficiencies are expected to maintain the attractiveness of this technique.

Electrospinning produces a 3D open porous structure which approximates the structure of mucosa. Natural materials (e.g., collagen or chitosan) to different synthetic materials, such as different types of polylactones, can be electrospun. With a combination of synthetic and biological biomaterials, it is possible to create hybrid materials with the properties of each. For fabrication of tissue-engineered urethra, Zhang et al. [49] used the co-axial electrospinning technique to fabricate a nanofiber scaffold composed of collagen type I and poly(L-lactide-co-caprolactone) (P(LLA-CL)) with the aim of structurally and morphologically mimicking the extracellular matrix (ECM). This combination led to a material with satisfactory biomechanical properties (e.g., programmed biodegradation, tensile strength, strain at break, etc.). Compared to the blending method, core–shell co-axial electrospinning can also protect biologically active compounds during fabrication. The authors thus used this technique to load their collagen/P(LLA-CL) scaffolds with the Wnt pathway inhibitor ICG-001. Such nanofiber scaffolds with high mechanical properties supported urethra reconstruction in a rabbit model of urethra defect. It was noted that these ICG-001-releasing scaffolds significantly inhibited ECM expression of fibroblasts, supporting the idea that these scaffolds have an anti-fibrosis effect in vitro. 

Electrospinning technology was used to prepare poly(L-lactide)/poly(ethylene glycol) (PLLA/PEG) fibrous scaffolds with various PEG fractions in a study by Lv et al. [50]. Human amniotic mesenchymal stem cells (hAMSCs) adhered and proliferated well on the scaffolds. After seeding with hAMSCs, substitutes were implanted in a rabbit model of long-segment urethral defects. Results from the morphology and tissue reconstruction evaluation, including complication incidence among the animals, showed that PLLA/PEG scaffolds combined with hAMSCs led to the better repair of urethral defects than PLLA/PEG scaffolds without hAMSCs. This is evidence that cells by their paracrine response positively affect the regeneration processes. 

A novel electrospun silk fibroin matrix was prepared by Xie et al. [51]. The structure was assessed by scanning electron microscopy and a porosity test. Canine urothelial cells were seeded upon the structure to obtain a tissue-engineered substitute. The urothelial cells grew on the material and showed good biocompatibility with the stretched silk fibroin matrices. 

Wei et al. [52] blended polycaprolactone (PCL), collagen, and silk fibroin to prepare high-performance nanofiber scaffolds. They inoculated oral keratinocytes cultured in vitro on such scaffolds to obtain tissue-engineered mucosa for urethral reconstruction. The cells adhered and proliferated well on the material, had uniform morphology, and grew into the pores.

## 5. Cell Sheet Engineering

Zhou et al. [53] reported the fabrication of novel tissue-engineered bionic urethrae using cell sheet technology that were prepared using adipose-derived stem cells, oral mucosal epithelial cells, and oral mucosal fibroblasts. The cell sheets were hierarchically tubularized to form 3 layer tissue-engineered urethrae and labeled by ultra-small super-paramagnetic iron oxide (USPIO). Such implants were tested in a canine model; first, the bionic urethrae were transplanted subcutaneously for 3 weeks to promote vascularization and biomechanical strength. Then 2 cm lengths of tubularized penile urethra were replaced by the tissue-engineered bionic urethra. Histological analysis showed that the grafts retained their 3 layer architecture, including an epithelial layer, a fibrous layer, and a myoblast layer. At 3 months after urethral replacement, the new tissue was morphologically and functionally comparable to normal urethral tissue. It is known that urethral diseases often affect all urethral layers. The significance of this study is that the full-thickness defect of the urethral wall may be restored via cell sheet technology.

The urethra is a dynamic organ in the human body that has to be resistant to intraluminal pressure during urination. This is the reason why characteristics such as elasticity and folding/unfolding should be taken in account while engineering a urethral scaffold. This issue was investigated in the following study. A new technique for making tubular collagen scaffolds made use of compression of fibrillar collagen around a star-shaped mandrel to mimic the characteristic folds in the urethral lumen [33]. By in situ fixation with a star-shaped mandrel using 3D-printed clamps and cytocompatible carbodiimide crosslinking, a shape recovery effect was introduced. This represents a new type of collagen I based tubular scaffold with intrinsic radial elasticity. Subsequently, human epithelial cells seeded on the luminal side of the scaffold adhered well and were compatible with voiding dynamics in a bioreactor. Moreover, cells created several layers which are important for obtaining sufficient barrier function of the wall.

Finally, we will mention here a study by Cattan et al. [54] in which the authors developed a human tissue-engineered tubular genitourinary graft (TTGG) composed of human dermal fibroblasts and human urothelial cells without exogenous scaffolding. A bioreactor was used to investigate the effects of in vitro mechanical stimuli on the functional and morphological properties of this graft. Under dynamic conditions, the graft showed a well-established layered urothelium and basement membrane formation, which was not observed in static culture. Mechanical stimuli in vitro led to urothelium differentiation with morphological and functional properties equivalent to a native urethra. The authors described the advantages of TTGG as a cell-living graft with an extracellular matrix which could be easier to adapt when used in vivo; the graft had satisfactory mechanical properties and was suturable, which is crucial for surgical handling. The barrier function of TTGG was also demonstrated. This study proved the importance of mechanical stimuli on terminal differentiation and stratification of human urothelial cells under in vitro conditions. 

## 6. Bioprinting

**Figure 1:**
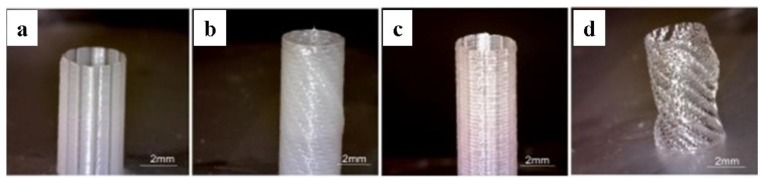
Examples of different tubular structures fabricated by 3D bioprinting. (**a**) Tubular structure with columnar design from PCL; (**b**) Tubular structure with spiral design from PCL; (**c**) Tubular structure with columnar design from PCL/P(LLA-CL) (50:50); (**d**) Tubular structure with spiral design from PCL/P(LLA-CL) (50:50).

The method of 3D bioprinting (Figure 1) is the newest strategy for urethral reconstruction. Bioprinting can be considered as an additive manufacturing technique where cells and biomaterials, referred to as bioink, are deposited simultaneously. This approach includes the incorporation of cells, growth factors, and biomaterial in a single step [55]. With the assistance of image data, bioprinting might also be applied to producing replacement tissues for defective organs according to the specific, practical conditions of different patients. This possibility arises from the technique’s ability to process multiple biomaterials and cell types simultaneously and to print a structure directly from digital codes in a computer file [56]. 

With regard to bioprinting, consideration of the bioinks to be used must take in account the state of biomaterials and cells not only during the period of early growth and regeneration, but also consider the possible long-term changes of bioprinted construct properties caused by cell proliferation, migration, and interaction with hydrogel material (e.g., cell traction, enzymatic degradation, matrix remodeling). There are also different ways for cells to proliferate inside the hydrogel constructs, and these differences in growth are not only attributed to the cell type, but also to the properties of the hydrogels, such as porosity, stiffness, and, most importantly, the presence of ligands facilitating cell attachment [57,58,59].

Zhang et al. [60] used 3D bioprinting technology to fabricate a cell-laden urethra in vitro with different polymer types and structural characteristics. They chose the known biocompatible polymers PCL and P(LLA-CL) and used a spiral scaffold design to mimic the structure and mechanical properties of the natural urethra of rabbits. A tubular scaffold was formed using an integrated bioprinting system where urothelial cells and smooth muscle cells were applied on both the inner and outer layer of the scaffolds separately within the cell-laden hydrogel. These pioneering results give a strong foundation for further study of 3D bioprinting of the urethrae. 

The biofabrication of multilayered tubular tissues or organs with cellular heterogeneity, such as blood vessels, trachea, intestine, colon, ureter, and urethra, remains a challenge for bioprinting technology. Pi et al. [61] recently presented a promising multichannel coaxial extrusion system (MCCES) for microfluidic bioprinting of circumferential multilayered tubular tissues, using special bioinks with gelatin methacryloyl, alginate, and eight-arm poly(ethylene glycol) acrylate with a tripentaerythritol core. The perfusable cannular constructs can be changed from a monolayer to triple layers along the length of a bioprinted tube. The bioprinted tubes demonstrated biofunctionality as assessed by cell viability, proliferation, and differentiation using relevant cell types. For bioprinted cannular urothelial tissue constructs, human urothelial cells and human bladder smooth muscle cells were used. For vascular tissue substitutes, human umbilical vein endothelial cells, and human smooth muscle cells were used. These bioprinted cannular tissues were actively perfused in a bioreactor to promote growth and proliferation of the seeded cell types. This technique results in the fabrication of tunable and perfusable multilayered tissues and is a fundamental step toward creating human cannular tissues.

## 7. Concluding Remarks

The current management of urethral strictures involves a variety of surgical techniques. Choosing the appropriate one depends on the length of the urethral stricture, on its location, as well as on the experiences of the surgeon. It is difficult to say which technique is superior. For example, buccal mucosa urethroplasty is often used to manage long and complex urethral defects, although there are frequent complications from the donor sites. Regenerative medicine and tissue engineering may provide an alternative for these urethral grafts for both pediatric and adult patients. In the context of degradable/regenerative scaffolds for urethral reconstruction, our review describes various types of biomaterials and manufacturing techniques. Cell-seeded scaffolds showed promising results for the management of long and complex urethral strictures. Based on studies using these scaffolds, several requirements have to be met. The supporting matrix (scaffold) has to be biocompatible as well as biodegradable with appropriate structural and mechanical properties (e.g., elasticity). Moreover, appropriate cell type has to be chosen for seeding, as well. Several cell types are present in the native urethra. Most of the studies used autologous urothelial cells, which can provide a sufficient barrier function against urine and smooth muscle cells. These can also provide the necessary tissue elasticity. Sufficient barrier function is crucial for the satisfactory functioning of the neo-urethra. Any leakage of the urine into the urethral wall triggers pro-stricture processes. There are still some issues that need to be addressed before these scaffold systems meet all the clinical requirements. First, there can be a long period before the scaffold system would be functional after placed in vivo. Other issues include the difficulty of seeding the entire scaffold homogeneously, inability to spatially distribute multiple cell types, and poor control of the scaffold micro-architecture. On the other hand, this approach presents minimal intervention for the patient. However, only a few studies described the application of the tissue-engineered tubular scaffolds in patients, with most done in animal models. Therefore, in our opinion, the problem of transferring these experiments into clinical medicine means that we still do not have an ideal and guaranteed method for making tissue-engineered scaffolds for urethral reconstruction. Issues such as vascular and nerve supply, proper barrier function, and the effects of metabolite degradation products are still not fully solved. Even if the scaffold is made from autologous tissue, the host immune response is unpredictable. However, technologies such as electrospinning or bioprinting have the potential to make engineered scaffolds that approximate the native structure, composition, and activity to enhance the process of regeneration. Finally, it can be concluded that tissue engineering and regenerative medicine have great potential for the management of the urethral strictures. However, more research still needs to be done to transfer experimental outcomes into clinical medicine. 

## Figures and Tables

**Table 1:** Selected biological scaffolds used as tubular urethral substitutes.

Material	Results	Reference
human amniotic membrane	confirmed potential, concern about the lack of standardized preparation protocol, storage, and mechanical properties	[22]
tubular gelatin scaffold loaded with EGF and MMC	inhibitory potential of scar formation	[31]
seeded bladder submucosa	successful repair of a long urethral defect in a canine model	[24]
unseeded bladder submucosa	ability to repair short (0.5 cm) urethral defects; long defects (up to 3 cm) were not repaired, increased deposition of collagen and fibrosis detected	[25]
collagen scaffold loaded with CB-VEGF	better epithelization, revascularization and smooth muscle regeneration detected	[26]
double-layered high-density collagen gel tubes	the regenerative potential of gel tubes observed (animal model); however, 20% of animals developed complications	[27]
silk fibroin	good biodegradation properties	[35]
modified silk fibroin/keratin films with oxygen-generating substance and calcium peroxide	observed enhanced regenerative potential	[36]

EGF—epidermal growth factor, MMC—mitomycin C, CB-VEGF—collagen-binding vascular endothelial growth factor.

**Table 2:** Selected biodegradable synthetic scaffolds used as tubular urethral substitutes.

Material	Results	Reference
the graft copolymer of PLA	better mechanical properties when compared to PLA homopolymer	[37]
PLGA	autologous tissue-engineered urethras applied in 5 boys remained functional up to 6 years’ follow-up	[16]
PLGA	observed potential for tissue engineering of buccal mucosa for urethral repair application	[39]
PEUs	estimated satisfactory biological properties; possible saturation with urethral adhesive proteins	[40,41]

PLA—polylactic acid; PLGA—poly(lactic-co-glycolic acid); PEUs—polyurethanes.
